# The Language of Flowers

**Published:** 1877-03

**Authors:** 


### THE LANGUAGE OF FLOWERS.

Nature has "painted the lily" and tinted even the humblest flower by the wayside with a skill that the highest art can never approach. She has given them a language which may be read and comprehended by all ages and conditions of mankind, and whose influence is always for good.

There is no home that so cheers the heart of every one who approaches it as the neat cottage with its well-kept grounds and a profusion of choice plants and flowersarranged and blended with a symmetry that pleases the eye.

We are certain that the full measure of peace, happiness, and refinement dwell in that home.

Every one, and particularly children, should cultivate a taste for flowers. There are few things that bring so much real pleasure, and nothing so desirable, that cost so little. James Vick, of Rochester, N. Y., is to be envied probably more than any other gentleman in the State. He lives in a sort of earthly Paradise, with rarest flowers for his companions at all seasons. In addition to the pleasure derived from such beautiful surroundings, he contributes to the adornment of thousands of homes and to the happiness of all their occupants.

There is no person but can take care of a few plants. There is hardly a woman in France, whatever her condition in life, but has a row of flower-pots in the window. Mr. Vick publishes quarterly a beautiful illustrated book called the "Floral Guide" at twenty-five cents a year.

We advise our readers to send for it, make their selections, and send for plants and seeds before the season is too far advanced for planting.

---

An Editor's Sanctum is usually a most uninviting place. Small, dark, and dingy, strewn with newspapers, furnished with a rickety pine table, a three-legged stool, and a spittoon. This is the workshop of the oracle who shapes and directs "public opinion," and who is supposed to know everything. An unexpected event occurs, and within a few hours an exhaustive article appears in the newspapers giving a history of all similar events for ages. How are the facts obtained? The popular belief is that they are supplied from the vast storehouse of the editor's memory. Those who have been behind the scenes know that editors of great newspapers are about as wise as other educated men, and not a whit more so. Like all skillful workmen, they require the best of tools to work with. Deprive them of "Zell's Encyclopedia" and "Webster's Unabridged Dictionary," and their articles would be meager in facts and defective in style.

The genius of an editor depends more upon the skill with which he collates and applies the facts gathered from books when required than from anything his memory could furnish, though a giant in intellect.

### *FIRE ON THE HEARTH.*

Several hundred persons have called at our office and examined the stove which we bought of the Open Stove Ventilating Company, 107 Fulton Street, last fall. Everybody seems pleased with it, but nobody can begin to comprehend all its excellencies until they have lived with it awhile. It has been remarked that "you never know a person until you have summered him and wintered him." This is, perhaps, true of a man or a woman, since the best or the worst qualities may manifest themselves under extremes of heat or cold. We have known people who were very disagreeable companions when perspiring, and others who were cross and wretched in very cold weather. But we will be satisfied with such testimony as may be uttered in the winter only, by a stove designed for heating, without reference to what it may say when stowed away for the summer in some closet or cellar. Now, we have lived with our "Fire on the Hearth" four good months, beginning with the last week in October, and covering some pretty cold weather. We can only say that it has worked to a charm. In the coldest days it has kept us comfortable, and in the warmer ones it has diffused just the pleasant temperature needed to make us contented to stay indoors. Besides, it has ventilated our room and kept the air always sweet and wholesome. It's a great institution, durable and economical.

---

Colton's Atlas.—We have recently had an opportunity of examining the latest edition of Colton's General Atlas of the World, and have been struck with its completeness and accuracy. Our first thought was to subject it to rigid scrutiny with a view to determining how truthfully it described the surveys, the railroads, and the explorations which have been pushed forward so energetically in our Western Territories during the past few years. Our scrutiny justifies us in asserting that there is nothing omitted in this direction. The very important work in the way of exploration, by the expeditions of Powell and Hayden and Wheeler, is fully noted, as are the various railroads constructed and projected. The information thus conveyed is of great importance to that very large proportion of our people who are looking with growing interest to that remarkable region from which are destined to spring the populous and wealthy States of the future. The maps comprised in this Atlas are 212 in number, illustrative of the geography, topography, population, and condition of the globe. It is a work which is not only not excelled in the world, but which we believe to be unapproached in accuracy and completeness. No scholar can well do without it, and no father of boys can afford to allow them to grow up unaided by its teachings.

---

Ormon's Magic Cleanser.—In our Journal for March, 1876, we spoke favorably of an article which we had tested for the removal of spots and stains from clothing. We were led to try it by our tailor, who confided to us the fact that all the best city tailors were in the habit of employing the fluid alluded to to cleanse and freshen soiled clothes. Everybody knows how hard it is to keep clothes, especially coat and vest fronts, from becoming soiled, and how nearly useless soaps and benzines are in the removal of such defacements. With this article there is not much trouble in keeping one's clothing neat and fresh and free from spots. It is now sold by the Health Food Company and by Macy in New York, and by Loesser in Brooklyn. The office of the proprietor, Mr. H. Wiswell, is with Lipe, Nearing & Co., 54 Lispenard Street, New York.

